# 1-(4-Meth­oxy­phen­yl)-2-[4-(tri­fluoro­meth­yl)phen­yl]-1*H*-phenanthro[9,10-*d*]imidazole

**DOI:** 10.1107/S1600536813019351

**Published:** 2013-07-20

**Authors:** T. Mohandas, R. Sathishkumar, J. Jayabharathi, A. Pasupathi, P. Sakthivel

**Affiliations:** a Shri Angalamman College of Engineering and Technology, Siruganoor, Tiruchirappalli 621 105, India; b Department of Chemistry, Annamalai University, Annamalainagar, Chidambaram, India; c Department of Chemistry, Urumudhanalakshmi College, Tiruchirappalli 620 019, India; d Department of Physics, Urumudhanalakshmi College, Tiruchirappalli 620 019, India

## Abstract

In the title compound, C_29_H_19_F_3_N_2_O, a phenanthroline-fused imidazole tetra­cyclic system, the fused benzene rings deviate slightly from the central ring and make dihedral angles with this ring of 3.47 (8) and 3.05 (8)°. The tri­fluoro­methyl­phenyl ring is roughly coplanar with the phenanthroline-fused imidazole tetra­cyclic system [dihedral angle = 11.02 (6)°], while the meth­oxy­phenyl ring is almost perpendicular [dihedral angle = 87.65 (6)°]. There are intra­molecular C—H ⋯π inter­actions involving the meth­oxy­phenyl ring and the central phenanthroline ring, as well as an inter­molecular C—H⋯π inter­action involving the phenanthroline ring. In addition, there is an inter­molecular π–π inter­action involving the central phenanthroline ring and the tri­fluoro­methyl­phenyl ring [centroid–centroid distance = 3.685 (2) Å], as well as C—H⋯N inter­actions linking the mol­ecules into dimers.

## Related literature

For background to the supra­molecular architecture of phenanthroline compounds, see: Lehn (1995 ▶). For metal sensors, see: Walters *et al.* (2000 ▶). For mol­ecular electronics, see: Peng *et al.* (1997 ▶). For photo sensitizers see: Hara *et al.* (2001 ▶). For a related structure, see: Sathishkumar *et al.* (2013 ▶).
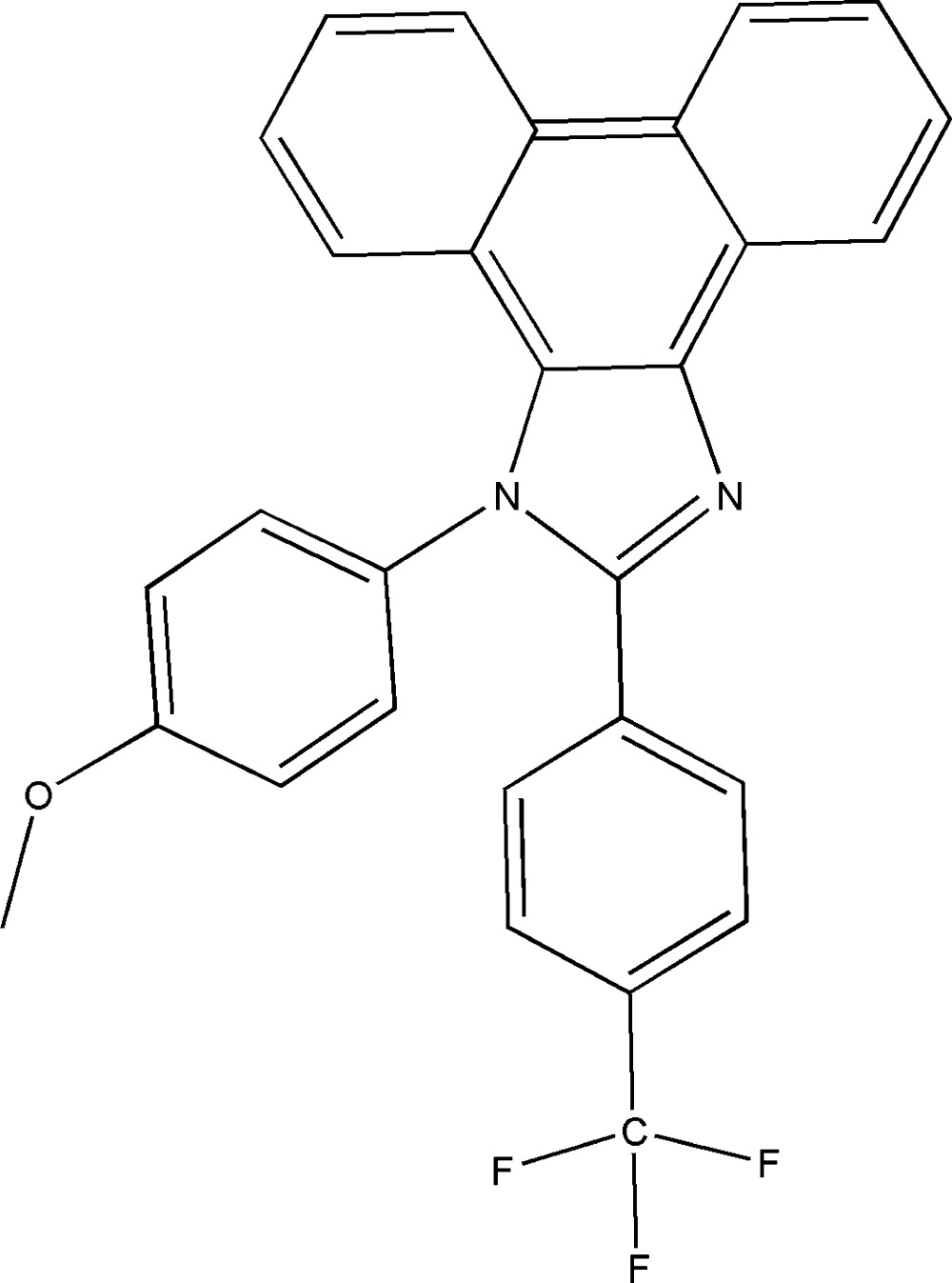


.

## Experimental

### 

#### Crystal data

C_29_H_19_F_3_N_2_O
*M* *_r_* = 468.46
Monoclinic, 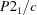

*a* = 11.7063 (9) Å
*b* = 20.2301 (16) Å
*c* = 9.5419 (8) Å
β = 99.725 (2)°
*V* = 2227.2 (3) Å^3^
*Z* = 4
Mo *K*α radiation
μ = 0.10 mm^−1^
*T* = 173 K
0.32 × 0.29 × 0.25 mm

#### Data collection

Bruker Kappa APEXII CCD diffractometer
Absorption correction: multi-scan (*SADABS*; Bruker, 2008 ▶) *T*_min_ = 0.956, *T*_max_ = 0.999
24449 measured reflections
5128 independent reflections
3511 reflections with *I* > 2σ(*I*)
*R*_int_ = 0.032

#### Refinement

*R*[*F* ^2^ > 2σ(*F* ^2^)] = 0.045
*wR*(*F* ^2^) = 0.124
*S* = 1.01
5128 reflections
317 parameters
H-atom parameters constrained
Δρ_max_ = 0.18 e Å^−3^
Δρ_min_ = −0.24 e Å^−3^

### 

Data collection: *APEX2* (Bruker, 2008 ▶); cell refinement: *APEX2* and *SAINT* (Bruker, 2008 ▶); data reduction: *SAINT*; program(s) used to solve structure: *SHELXS97* (Sheldrick, 2008 ▶); program(s) used to refine structure: *SHELXL97* (Sheldrick, 2008 ▶); molecular graphics: *ORTEP-3* (Farrugia, 2012 ▶); software used to prepare material for publication: *PLATON* (Spek, 2009 ▶).

## Supplementary Material

Crystal structure: contains datablock(s) global, I. DOI: 10.1107/S1600536813019351/bv2223sup1.cif

Structure factors: contains datablock(s) I. DOI: 10.1107/S1600536813019351/bv2223Isup2.hkl

Additional supplementary materials:  crystallographic information; 3D view; checkCIF report

## Figures and Tables

**Table 1:** Hydrogen-bond geometry (Å, °) *Cg*1 and *Cg* are the centroids of the methoxyphenyl and phenthroline rings, repsectively.

*D*—H⋯*A*	*D*—H	H⋯*A*	*D*⋯*A*	*D*—H⋯*A*
C28—H28⋯N2^i^	0.95	2.55	3.402 (2)	150
C3—H3⋯*Cg*1	0.95	2.86	3.719 (2)	154
C10—H10⋯*Cg*2^ii^	0.95	2.69	3.419 (2)	136
C17—H17⋯*Cg*1	0.95	2.74	3.6042 (2)	154

Symmetry codes: (i) 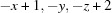; (ii) 
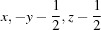
.

**Fig. 1.**
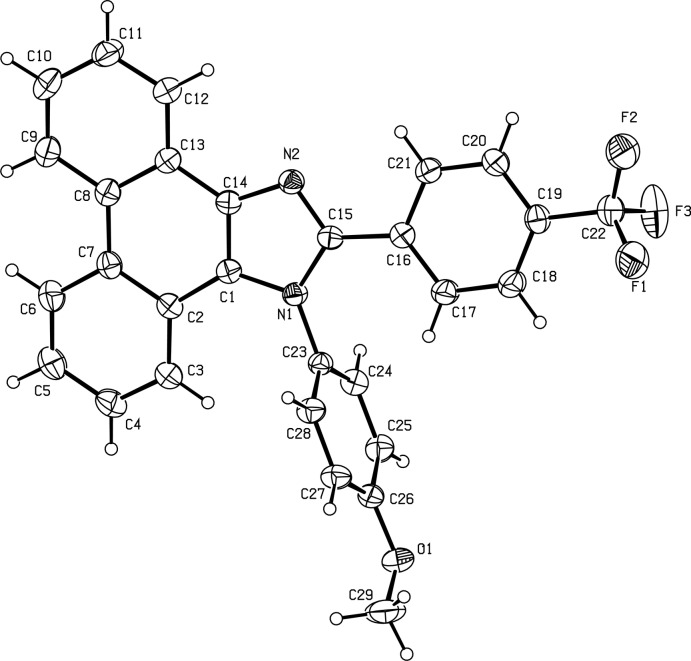
The molecular structure and labelling scheme for (I) with displacement ellipsoids for non-H atoms are drawn at the 30% probability level.

**Fig. 2.**
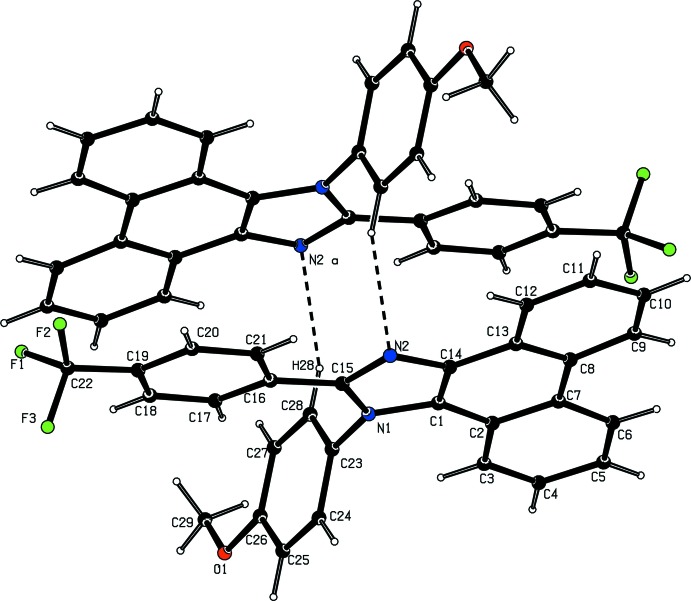
A diagram showing the C-H···N out of plane π interactions linking molecules into dimers.A packing diagram for (I) is shown.

## supplementary crystallographic information

### Comment

The large variety of complexes based on phenanthroline or polypyridyl derivatives allows the formation of many different molecular systems with various applications ranging from metallo-supramolecular chemistry (Lehn, 1995), metal sensors (Walters *et al.*, 2000), molecular electronics (Peng *et al.*, 1997), and photo sensitizers (Hara *et al.*, 2001). Therefore, in the recent years the preparation of phenanthroimidazoles has gained great attention.

In this phenanthroline triclinic ring system, the phenyl rings on either end are slightly deviated from the central ring and make the dihedral angles with this ring of 3.47 (8)° and 3.05 (8)° respectively. The trifluromethyl phenyl ring is almost coplanar with the phenanthroline fused imidazole tetracyclic system (dihedral angle of 11.02 (6)°) while methoxy phenyl ring is almost perpendicular (dihedral angle of 87.65 (6)°).

The maximum deviation of C9 atom from the mean plane of the tetracyclic phenantherene fused imidazole ring is 0.078 (2)° and that of C29 atom from the methoxy phenyl ring is -0.043 (3)°. The C22 atom of the trifluromethyl phenyl ring is deviated by -0.040 (2)°.

There are intramolecular C3—H3 ···π and C17—H17···π (methoxyphenyl ring system) interactions as well as an intermolecular C10—H10···π interaction involving the phenanthroline ring (at the symmetry code *x*, 1/2 - y, 1/2 + *z*). There is an intermolecular π–π interaction involving the central ring phenanthroline ring and the trifluoromethyl phenyl ring (Cg···Cg 3.685 Å, 1-x, -y, 2-z). In addition there C—H···N out-of-plane interactions linking the molecules into dimers as shown in Fig. 2.

### Experimental

A mixture of phenanthrene-9,10-dione (1.0 g,4.8 mmol), ammonium acetate (1.48 g,19.2 mmol), 4-trifluromethylbenzaldehyde(0.83 g,4.8 mmol) and 4-methoxyaniline (2.95 g,24 mmol) have been refluxed in ethanol (20 mL) at 80°C. The reaction was monitored by TLC and purified by column chromatography using petroleum ether:ethyl acetate (9:1) as the eluent.Yield: 0.74 g (51%)

### Refinement

All the hydrogen atoms were geometrically fixed and allowed to ride on their parent atoms with C—H= 0.93 - 0.97 Å,and *U*_iso_(H) = 1.3U_eq_(C)

### Crystal data

**Table:**

C_29_H_19_F_3_N_2_O	*F*(000) = 968
*M**_r_* = 468.46	*D*_x_ = 1.397 Mg m^−^^3^
Monoclinic, *P*2_1_/*c*	Mo *K*α radiation, λ = 0.71073 Å
Hall symbol: -P 2ybc	Cell parameters from 5131 reflections
*a* = 11.7063 (9) Å	θ = 1.8–27.6°
*b* = 20.2301 (16) Å	µ = 0.10 mm^−^^1^
*c* = 9.5419 (8) Å	*T* = 173 K
β = 99.725 (2)°	Block, colourless
*V* = 2227.2 (3) Å^3^	0.32 × 0.29 × 0.25 mm
*Z* = 4	

### Data collection

**Table:**

Bruker Kappa APEXII CCD diffractometer	5128 independent reflections
Radiation source: fine-focus sealed tube	3511 reflections with *I* > 2σ(*I*)
Graphite monochromator	*R*_int_ = 0.032
Ω and φ scan	θ_max_ = 27.6°, θ_min_ = 2.4°
Absorption correction: multi-scan (*SADABS*; Bruker, 2008)	*h* = −15→14
*T*_min_ = 0.956, *T*_max_ = 0.999	*k* = 0→26
24449 measured reflections	*l* = 0→12

### Refinement

**Table:**

Refinement on *F*^2^	Primary atom site location: structure-invariant direct methods
Least-squares matrix: full	Secondary atom site location: difference Fourier map
*R*[*F*^2^ > 2σ(*F*^2^)] = 0.045	Hydrogen site location: inferred from neighbouring sites
*wR*(*F*^2^) = 0.124	H-atom parameters constrained
*S* = 1.01	*w* = 1/[σ^2^(*F*_o_^2^) + (0.0514*P*)^2^ + 0.6328*P*] where *P* = (*F*_o_^2^ + 2*F*_c_^2^)/3
5128 reflections	(Δ/σ)_max_ < 0.001
317 parameters	Δρ_max_ = 0.18 e Å^−^^3^
0 restraints	Δρ_min_ = −0.24 e Å^−^^3^

### Special details

**Table:**

Geometry. All esds (except the esd in the dihedral angle between two l.s. planes) are estimated using the full covariance matrix. The cell esds are taken into account individually in the estimation of esds in distances, angles and torsion angles; correlations between esds in cell parameters are only used when they are defined by crystal symmetry. An approximate (isotropic) treatment of cell esds is used for estimating esds involving l.s. planes.
Refinement. Refinement of F^2^ against ALL reflections. The weighted R-factor wR and goodness of fit S are based on F^2^, conventional R-factors R are based on F, with F set to zero for negative F^2^. The threshold expression of F^2^ > 2sigma(F^2^) is used only for calculating R-factors(gt) etc. and is not relevant to the choice of reflections for refinement. R-factors based on F^2^ are statistically about twice as large as those based on F, and R- factors based on ALL data will be even larger.

### Fractional atomic coordinates and isotropic or equivalent isotropic displacement parameters (Å^2^)

**Table:**

	*x*	*y*	*z*	*U*_iso_*/*U*_eq_	
F1	0.94082 (11)	−0.18352 (6)	0.83630 (17)	0.0945 (5)	
F2	1.03787 (11)	−0.09609 (7)	0.88946 (19)	0.1026 (5)	
F3	0.97313 (14)	−0.11886 (9)	0.67561 (17)	0.1112 (6)	
O1	0.24682 (11)	−0.21387 (6)	0.47694 (14)	0.0615 (4)	
N1	0.41440 (11)	0.01373 (6)	0.76779 (13)	0.0381 (3)	
N2	0.53956 (11)	0.08671 (6)	0.88301 (14)	0.0414 (3)	
C1	0.34962 (13)	0.06745 (7)	0.80056 (16)	0.0375 (3)	
C2	0.22709 (13)	0.08118 (8)	0.77095 (16)	0.0404 (4)	
C3	0.14290 (15)	0.04019 (9)	0.69130 (19)	0.0516 (4)	
H3	0.1656	−0.0009	0.6565	0.062*	
C4	0.02892 (16)	0.05872 (11)	0.6633 (2)	0.0625 (5)	
H4	−0.0268	0.0306	0.6092	0.075*	
C5	−0.00549 (16)	0.11848 (11)	0.7138 (2)	0.0674 (6)	
H5	−0.0846	0.1313	0.6938	0.081*	
C6	0.07410 (16)	0.15882 (10)	0.7921 (2)	0.0591 (5)	
H6	0.0489	0.1995	0.8260	0.071*	
C7	0.19225 (14)	0.14223 (8)	0.82452 (17)	0.0439 (4)	
C8	0.27615 (14)	0.18711 (8)	0.90664 (17)	0.0435 (4)	
C9	0.24350 (17)	0.24581 (9)	0.96904 (19)	0.0546 (4)	
H9	0.1637	0.2571	0.9580	0.066*	
C10	0.32367 (19)	0.28690 (9)	1.0449 (2)	0.0626 (5)	
H10	0.2989	0.3260	1.0861	0.075*	
C11	0.44104 (19)	0.27210 (9)	1.0624 (2)	0.0621 (5)	
H11	0.4964	0.3011	1.1146	0.075*	
C12	0.47650 (16)	0.21546 (8)	1.00403 (18)	0.0519 (4)	
H12	0.5568	0.2053	1.0155	0.062*	
C13	0.39540 (14)	0.17238 (7)	0.92748 (16)	0.0410 (4)	
C14	0.42898 (13)	0.11102 (7)	0.87125 (16)	0.0387 (3)	
C15	0.52887 (13)	0.02806 (7)	0.82138 (16)	0.0380 (3)	
C16	0.63143 (13)	−0.01355 (7)	0.81555 (16)	0.0388 (3)	
C17	0.62932 (15)	−0.07882 (8)	0.76912 (19)	0.0505 (4)	
H17	0.5571	−0.1000	0.7379	0.061*	
C18	0.73102 (16)	−0.11314 (9)	0.7679 (2)	0.0543 (4)	
H18	0.7280	−0.1577	0.7360	0.065*	
C19	0.83675 (14)	−0.08351 (8)	0.81246 (18)	0.0467 (4)	
C20	0.84057 (15)	−0.01934 (9)	0.8612 (2)	0.0533 (4)	
H20	0.9131	0.0013	0.8938	0.064*	
C21	0.73894 (15)	0.01501 (8)	0.86267 (19)	0.0494 (4)	
H21	0.7426	0.0592	0.8967	0.059*	
C22	0.94602 (17)	−0.11998 (10)	0.8042 (2)	0.0606 (5)	
C23	0.36937 (13)	−0.04529 (7)	0.69415 (16)	0.0374 (3)	
C24	0.36221 (14)	−0.04960 (8)	0.54819 (17)	0.0434 (4)	
H24	0.3855	−0.0135	0.4960	0.052*	
C25	0.32095 (15)	−0.10681 (8)	0.47918 (18)	0.0482 (4)	
H25	0.3165	−0.1102	0.3791	0.058*	
C26	0.28607 (13)	−0.15923 (8)	0.55452 (18)	0.0450 (4)	
C27	0.29213 (15)	−0.15426 (8)	0.70017 (19)	0.0502 (4)	
H27	0.2676	−0.1901	0.7523	0.060*	
C28	0.33403 (15)	−0.09708 (8)	0.76958 (18)	0.0460 (4)	
H28	0.3384	−0.0936	0.8696	0.055*	
C29	0.2023 (2)	−0.26684 (12)	0.5502 (3)	0.0963 (9)	
H29A	0.1761	−0.3025	0.4830	0.144*	
H29B	0.1369	−0.2510	0.5929	0.144*	
H29C	0.2633	−0.2834	0.6251	0.144*	

### Atomic displacement parameters (Å^2^)

**Table:**

	*U*^11^	*U*^22^	*U*^33^	*U*^12^	*U*^13^	*U*^23^
F1	0.0725 (8)	0.0575 (8)	0.1538 (14)	0.0215 (6)	0.0206 (9)	0.0119 (8)
F2	0.0504 (7)	0.0934 (10)	0.1529 (14)	0.0193 (7)	−0.0148 (8)	−0.0329 (9)
F3	0.0985 (11)	0.1497 (14)	0.0970 (11)	0.0515 (10)	0.0497 (9)	0.0126 (10)
O1	0.0598 (8)	0.0564 (8)	0.0657 (8)	−0.0134 (6)	0.0028 (6)	−0.0200 (6)
N1	0.0384 (7)	0.0357 (6)	0.0391 (7)	−0.0013 (5)	0.0033 (5)	−0.0015 (5)
N2	0.0398 (7)	0.0414 (7)	0.0417 (7)	−0.0010 (6)	0.0033 (6)	−0.0023 (6)
C1	0.0414 (8)	0.0365 (8)	0.0347 (8)	−0.0008 (6)	0.0067 (6)	0.0039 (6)
C2	0.0397 (8)	0.0431 (8)	0.0383 (8)	−0.0010 (7)	0.0062 (6)	0.0059 (7)
C3	0.0422 (9)	0.0547 (10)	0.0570 (11)	−0.0020 (8)	0.0058 (8)	−0.0037 (8)
C4	0.0418 (10)	0.0735 (13)	0.0690 (13)	−0.0058 (9)	0.0002 (9)	−0.0066 (10)
C5	0.0391 (10)	0.0775 (14)	0.0831 (15)	0.0087 (10)	0.0035 (9)	0.0001 (12)
C6	0.0470 (10)	0.0600 (11)	0.0709 (13)	0.0087 (9)	0.0118 (9)	−0.0003 (10)
C7	0.0431 (9)	0.0467 (9)	0.0432 (9)	0.0031 (7)	0.0112 (7)	0.0065 (7)
C8	0.0504 (9)	0.0427 (8)	0.0392 (8)	0.0019 (7)	0.0127 (7)	0.0045 (7)
C9	0.0592 (11)	0.0505 (10)	0.0568 (11)	0.0083 (9)	0.0175 (9)	−0.0018 (8)
C10	0.0811 (14)	0.0501 (10)	0.0589 (12)	0.0058 (10)	0.0186 (10)	−0.0136 (9)
C11	0.0718 (13)	0.0532 (11)	0.0610 (12)	−0.0081 (10)	0.0102 (10)	−0.0159 (9)
C12	0.0549 (10)	0.0471 (9)	0.0536 (10)	−0.0038 (8)	0.0083 (8)	−0.0082 (8)
C13	0.0482 (9)	0.0392 (8)	0.0363 (8)	−0.0009 (7)	0.0086 (7)	0.0016 (6)
C14	0.0402 (8)	0.0379 (8)	0.0375 (8)	−0.0014 (7)	0.0054 (6)	0.0020 (6)
C15	0.0391 (8)	0.0395 (8)	0.0344 (8)	−0.0031 (7)	0.0033 (6)	0.0014 (6)
C16	0.0412 (8)	0.0412 (8)	0.0339 (8)	0.0003 (7)	0.0059 (6)	0.0029 (6)
C17	0.0427 (9)	0.0438 (9)	0.0625 (11)	−0.0007 (7)	0.0018 (8)	−0.0029 (8)
C18	0.0524 (10)	0.0424 (9)	0.0665 (12)	0.0042 (8)	0.0053 (9)	−0.0037 (8)
C19	0.0447 (9)	0.0482 (9)	0.0470 (9)	0.0067 (8)	0.0073 (7)	0.0049 (7)
C20	0.0404 (9)	0.0550 (10)	0.0629 (11)	0.0003 (8)	0.0040 (8)	−0.0046 (9)
C21	0.0458 (9)	0.0446 (9)	0.0562 (10)	−0.0001 (8)	0.0041 (8)	−0.0088 (8)
C22	0.0517 (11)	0.0564 (11)	0.0738 (14)	0.0082 (9)	0.0105 (10)	−0.0025 (10)
C23	0.0356 (8)	0.0366 (8)	0.0385 (8)	−0.0016 (6)	0.0023 (6)	−0.0017 (6)
C24	0.0452 (9)	0.0450 (9)	0.0388 (9)	−0.0016 (7)	0.0043 (7)	0.0057 (7)
C25	0.0504 (10)	0.0563 (10)	0.0355 (9)	0.0002 (8)	0.0003 (7)	−0.0043 (7)
C26	0.0357 (8)	0.0465 (9)	0.0503 (10)	−0.0025 (7)	0.0002 (7)	−0.0086 (7)
C27	0.0546 (10)	0.0447 (9)	0.0513 (10)	−0.0127 (8)	0.0091 (8)	0.0011 (8)
C28	0.0541 (10)	0.0470 (9)	0.0370 (8)	−0.0083 (8)	0.0081 (7)	−0.0012 (7)
C29	0.108 (2)	0.0749 (15)	0.112 (2)	−0.0536 (15)	0.0387 (16)	−0.0358 (14)

### Geometric parameters (Å, º)

**Table:**

F1—C22	1.325 (2)	C11—H11	0.9500
F2—C22	1.325 (2)	C12—C13	1.400 (2)
F3—C22	1.319 (2)	C12—H12	0.9500
O1—C26	1.3661 (19)	C13—C14	1.433 (2)
O1—C29	1.425 (3)	C15—C16	1.475 (2)
N1—C15	1.3819 (19)	C16—C21	1.389 (2)
N1—C1	1.3907 (19)	C16—C17	1.392 (2)
N1—C23	1.4395 (19)	C17—C18	1.380 (2)
N2—C15	1.3209 (19)	C17—H17	0.9500
N2—C14	1.371 (2)	C18—C19	1.376 (2)
C1—C14	1.372 (2)	C18—H18	0.9500
C1—C2	1.441 (2)	C19—C20	1.377 (2)
C2—C3	1.408 (2)	C19—C22	1.490 (2)
C2—C7	1.423 (2)	C20—C21	1.380 (2)
C3—C4	1.368 (2)	C20—H20	0.9500
C3—H3	0.9500	C21—H21	0.9500
C4—C5	1.386 (3)	C23—C28	1.374 (2)
C4—H4	0.9500	C23—C24	1.384 (2)
C5—C6	1.363 (3)	C24—C25	1.378 (2)
C5—H5	0.9500	C24—H24	0.9500
C6—C7	1.406 (2)	C25—C26	1.381 (2)
C6—H6	0.9500	C25—H25	0.9500
C7—C8	1.464 (2)	C26—C27	1.383 (2)
C8—C13	1.408 (2)	C27—C28	1.381 (2)
C8—C9	1.410 (2)	C27—H27	0.9500
C9—C10	1.366 (3)	C28—H28	0.9500
C9—H9	0.9500	C29—H29A	0.9800
C10—C11	1.388 (3)	C29—H29B	0.9800
C10—H10	0.9500	C29—H29C	0.9800
C11—C12	1.369 (2)		
			
C26—O1—C29	117.39 (15)	N1—C15—C16	127.66 (13)
C15—N1—C1	106.52 (12)	C21—C16—C17	117.66 (15)
C15—N1—C23	127.36 (12)	C21—C16—C15	116.69 (14)
C1—N1—C23	126.12 (13)	C17—C16—C15	125.64 (14)
C15—N2—C14	105.58 (13)	C18—C17—C16	120.74 (16)
C14—C1—N1	105.24 (13)	C18—C17—H17	119.6
C14—C1—C2	122.77 (14)	C16—C17—H17	119.6
N1—C1—C2	131.98 (14)	C19—C18—C17	120.69 (16)
C3—C2—C7	119.22 (15)	C19—C18—H18	119.7
C3—C2—C1	125.09 (15)	C17—C18—H18	119.7
C7—C2—C1	115.66 (14)	C18—C19—C20	119.41 (16)
C4—C3—C2	120.96 (17)	C18—C19—C22	120.21 (16)
C4—C3—H3	119.5	C20—C19—C22	120.36 (16)
C2—C3—H3	119.5	C19—C20—C21	119.95 (16)
C3—C4—C5	120.16 (18)	C19—C20—H20	120.0
C3—C4—H4	119.9	C21—C20—H20	120.0
C5—C4—H4	119.9	C20—C21—C16	121.52 (16)
C6—C5—C4	120.07 (18)	C20—C21—H21	119.2
C6—C5—H5	120.0	C16—C21—H21	119.2
C4—C5—H5	120.0	F3—C22—F2	105.49 (18)
C5—C6—C7	122.25 (18)	F3—C22—F1	105.03 (17)
C5—C6—H6	118.9	F2—C22—F1	106.03 (17)
C7—C6—H6	118.9	F3—C22—C19	112.60 (17)
C6—C7—C2	117.35 (16)	F2—C22—C19	113.69 (17)
C6—C7—C8	121.05 (16)	F1—C22—C19	113.25 (16)
C2—C7—C8	121.58 (14)	C28—C23—C24	120.45 (14)
C13—C8—C9	116.85 (16)	C28—C23—N1	119.65 (13)
C13—C8—C7	120.25 (14)	C24—C23—N1	119.91 (13)
C9—C8—C7	122.90 (16)	C25—C24—C23	119.38 (15)
C10—C9—C8	121.69 (18)	C25—C24—H24	120.3
C10—C9—H9	119.2	C23—C24—H24	120.3
C8—C9—H9	119.2	C24—C25—C26	120.45 (15)
C9—C10—C11	120.66 (17)	C24—C25—H25	119.8
C9—C10—H10	119.7	C26—C25—H25	119.8
C11—C10—H10	119.7	O1—C26—C25	116.18 (15)
C12—C11—C10	119.56 (18)	O1—C26—C27	123.97 (16)
C12—C11—H11	120.2	C25—C26—C27	119.85 (15)
C10—C11—H11	120.2	C28—C27—C26	119.76 (15)
C11—C12—C13	120.53 (18)	C28—C27—H27	120.1
C11—C12—H12	119.7	C26—C27—H27	120.1
C13—C12—H12	119.7	C23—C28—C27	120.10 (15)
C12—C13—C8	120.70 (15)	C23—C28—H28	120.0
C12—C13—C14	121.98 (15)	C27—C28—H28	120.0
C8—C13—C14	117.29 (14)	O1—C29—H29A	109.5
N2—C14—C1	111.17 (13)	O1—C29—H29B	109.5
N2—C14—C13	126.49 (14)	H29A—C29—H29B	109.5
C1—C14—C13	122.31 (14)	O1—C29—H29C	109.5
N2—C15—N1	111.48 (13)	H29A—C29—H29C	109.5
N2—C15—C16	120.86 (13)	H29B—C29—H29C	109.5
			
C15—N1—C1—C14	0.30 (16)	C8—C13—C14—C1	−0.5 (2)
C23—N1—C1—C14	179.48 (13)	C14—N2—C15—N1	0.87 (17)
C15—N1—C1—C2	179.13 (15)	C14—N2—C15—C16	−179.16 (13)
C23—N1—C1—C2	−1.7 (2)	C1—N1—C15—N2	−0.75 (16)
C14—C1—C2—C3	175.80 (16)	C23—N1—C15—N2	−179.92 (13)
N1—C1—C2—C3	−2.9 (3)	C1—N1—C15—C16	179.28 (14)
C14—C1—C2—C7	−2.3 (2)	C23—N1—C15—C16	0.1 (2)
N1—C1—C2—C7	179.08 (15)	N2—C15—C16—C21	−7.2 (2)
C7—C2—C3—C4	0.9 (3)	N1—C15—C16—C21	172.77 (15)
C1—C2—C3—C4	−177.09 (17)	N2—C15—C16—C17	171.79 (15)
C2—C3—C4—C5	−0.2 (3)	N1—C15—C16—C17	−8.2 (3)
C3—C4—C5—C6	−0.4 (3)	C21—C16—C17—C18	−1.2 (3)
C4—C5—C6—C7	0.2 (3)	C15—C16—C17—C18	179.86 (16)
C5—C6—C7—C2	0.6 (3)	C16—C17—C18—C19	−0.1 (3)
C5—C6—C7—C8	178.84 (18)	C17—C18—C19—C20	1.2 (3)
C3—C2—C7—C6	−1.1 (2)	C17—C18—C19—C22	−177.10 (18)
C1—C2—C7—C6	177.11 (15)	C18—C19—C20—C21	−1.1 (3)
C3—C2—C7—C8	−179.34 (15)	C22—C19—C20—C21	177.20 (17)
C1—C2—C7—C8	−1.2 (2)	C19—C20—C21—C16	−0.1 (3)
C6—C7—C8—C13	−174.43 (15)	C17—C16—C21—C20	1.3 (3)
C2—C7—C8—C13	3.8 (2)	C15—C16—C21—C20	−179.66 (16)
C6—C7—C8—C9	6.1 (2)	C18—C19—C22—F3	80.8 (2)
C2—C7—C8—C9	−175.70 (15)	C20—C19—C22—F3	−97.5 (2)
C13—C8—C9—C10	0.7 (3)	C18—C19—C22—F2	−159.34 (18)
C7—C8—C9—C10	−179.85 (17)	C20—C19—C22—F2	22.4 (3)
C8—C9—C10—C11	0.3 (3)	C18—C19—C22—F1	−38.2 (3)
C9—C10—C11—C12	−0.6 (3)	C20—C19—C22—F1	143.46 (19)
C10—C11—C12—C13	−0.2 (3)	C15—N1—C23—C28	92.07 (19)
C11—C12—C13—C8	1.3 (3)	C1—N1—C23—C28	−86.95 (19)
C11—C12—C13—C14	−176.70 (16)	C15—N1—C23—C24	−87.77 (19)
C9—C8—C13—C12	−1.5 (2)	C1—N1—C23—C24	93.20 (18)
C7—C8—C13—C12	179.04 (15)	C28—C23—C24—C25	−1.0 (2)
C9—C8—C13—C14	176.61 (14)	N1—C23—C24—C25	178.82 (14)
C7—C8—C13—C14	−2.9 (2)	C23—C24—C25—C26	0.5 (3)
C15—N2—C14—C1	−0.67 (17)	C29—O1—C26—C25	−175.49 (19)
C15—N2—C14—C13	177.31 (14)	C29—O1—C26—C27	4.5 (3)
N1—C1—C14—N2	0.23 (17)	C24—C25—C26—O1	−179.80 (15)
C2—C1—C14—N2	−178.74 (13)	C24—C25—C26—C27	0.2 (3)
N1—C1—C14—C13	−177.86 (13)	O1—C26—C27—C28	179.47 (16)
C2—C1—C14—C13	3.2 (2)	C25—C26—C27—C28	−0.6 (3)
C12—C13—C14—N2	−0.2 (2)	C24—C23—C28—C27	0.7 (2)
C8—C13—C14—N2	−178.24 (15)	N1—C23—C28—C27	−179.15 (15)
C12—C13—C14—C1	177.59 (15)	C26—C27—C28—C23	0.1 (3)

### Hydrogen-bond geometry (Å, º)

**Table:**

*D*—H···*A*	*D*—H	H···*A*	*D*···*A*	*D*—H···*A*
C28—H28···N2^i^	0.95	2.55	3.402 (2)	150
C3—H3···*Cg*1	0.95	2.86	3.719 (2)	154
C10—H10···*Cg*2^ii^	0.95	2.69	3.419 (2)	136
C17—H17···*Cg*1	0.95	2.74	3.6042 (2)	154

Symmetry codes: (i) −*x*+1, −*y*, −*z*+2; (ii) *x*, −*y*−1/2, *z*−1/2.
